# Exploring the pathogenesis of acute lung injury and its treatment through Traditional Chinese Medicine: a state-of-the-art review

**DOI:** 10.3389/fphar.2025.1592458

**Published:** 2025-07-31

**Authors:** Jiayun Wang, Zhiqiang Yan, Xinxin Zhang, Shun Wang, Liangbo Jiao, Binghua Zhu, Bo Tan, Aidong Yang

**Affiliations:** ^1^The Research Center for Traditional Chinese Medicine, Shanghai Institute of Infectious Diseases and Biosecurity, School of Traditional Chinese Medicine, Shanghai University of Traditional Chinese Medicine, Shanghai, China; ^2^Clinical Pharmacokinetic Laboratory, Shuguang Hospital Affiliated to Shanghai University of Traditional Chinese Medicine, Shanghai, China; ^3^ Department of Oncology, Baoshan Branch, Shuguang Hospital Affiliated to Shanghai University of Traditional Chinese Medicine, Shanghai, China; ^4^Department of Emergency & Intensive Care Unit, Shuguang Hospital Affiliated to Shanghai University of Traditional Chinese Medicine, Shanghai, China

**Keywords:** acute lung injury, pathogenesis, treatment, Traditional Chinese Medicine, bioactive compounds

## Abstract

Acute Lung Injury (ALI) is a severe and progressive condition characterized by hypoxic respiratory failure, often triggered by multiple contributing factors. It is associated with high morbidity and mortality rates and can advance to Acute Respiratory Distress Syndrome (ARDS) in severe cases. The pathogenesis of ALI involves a complex interplay of pathological mechanisms, including immune-inflammatory responses, disruption of the alveolar-capillary barrier, damage to mesenchymal stem cell organelles, metabolic dysregulation, ferroptosis, and alterations in gut microbiota. From the perspective of Traditional Chinese Medicine (TCM), the development of ALI is primarily attributed to the invasion of toxic pathogens, which result in lung dysfunction. TCM treatment strategies, which emphasize heat-clearing, detoxification, promoting blood circulation, and resolving stasis, have demonstrated promising clinical efficacy. This paper provides a comprehensive analysis of the pathogenesis of ALI and explores the therapeutic mechanisms of TCM compounds and bioactive monomers with potential therapeutic benefits. The goal is to establish a solid theoretical foundation for the clinical application of TCM in ALI treatment and to further validate its scientific rationale.

## 1 Introduction

ALI is characterized by decreased lung volume, reduced lung compliance, and an imbalance in the ventilation-perfusion ratio, which leads to clinical syndromes such as diffuse pulmonary interstitial edema and pulmonary edema. These conditions are primarily caused by lung infections (both bacterial and viral), as well as intrapulmonary or extrapulmonary factors, including lung contusion and sepsis, circulatory disorders associated with extracorporeal circulation, and immune system metabolic dysfunction. The underlying pathological mechanisms involve inflammation of the alveolar and pulmonary parenchyma, damage to the alveolar-capillary membrane, increased vascular permeability, and neutrophil recruitment (Dong et al., 2024; Mokrá, 2020). Clinically, ALI manifests as severe hypoxemia, changes in pulmonary function due to increased alveolar capillary membrane permeability, pulmonary edema, and respiratory failure.

From the perspective of Traditional Chinese Medicine (TCM), ALI is classified under the categories of “sudden asthma” and “out of the syndrome” with pathogenic factors including heat, toxins, phlegm, and blood stasis. These factors often result from external pathogenic invasions affecting the lungs, internal phlegm accumulation, and subsequent impairment of lung qi, leading to symptoms such as coughing, sputum production, constipation, and irritability. A summary of the common etiology, pathogenesis, and pathological characteristics of ALI from an integrated Chinese and Western medicine perspective is provided in Table 1.

**TABLE 1:** Etiology, Pathogenesis and pathological Characteristics of ALI from an Integrated Chinese and Western Medicine Perspective.

Etiological categorization	Specific causes	Pathogenesis in traditional Chinese medicine	Pathology in western medicine	Document source
Infectious agent	Bacteria, Virus	Pathogenic factors such as wind-cold or wind-heat enter the body through the mouth and nose, invading the lungs. This disrupts the lung’s functions of dispersing and descending qi, leading to qi stagnation and impaired distribution of body fluids. Over time, this results in the production of phlegm, which obstructs the lungs, further exacerbating respiratory dysfunction	Viral infections and bacterial proliferation in the lungs lead to the release of metabolites, which initiate inflammatory responses and cause subsequent tissue damage	McKelvey et al. (2024)
	Pyemia	Internal deficiency of vital qi, accumulation of toxic heat, obstruction of qi circulation, and the formation of blood stasis	The body becomes susceptible to external infections, resulting in an imbalanced inflammatory response and weakened immune function	Zeng (2019), Van der Poll et al. (2017)
Non-infectious Factor	Hemorrhagic shock induced by mechanical trauma	/	Pulmonary vascular protein leakage, neutrophil recruitment, and overexpression of proinflammatory factors, among other pathophysiological changes, lead to persistent and severe hypoxemia	Hao (2023)
The perfusion of the extracorporeal circulation system is compromised	Reperfusion following ischemic events	/	The imbalance between SOD and MDA in lung tissue led to the release and accumulation of oxygen free radicals within mitochondria, consequently increasing pulmonary vascular permeability and resulting in pulmonary edema, hypoxemia, and pulmonary hypertension	Shen et al. (2019)
Metabolic dysfunction within the immune system	Septicemia	The invasion of exogenous pathogens results in blood stasis and obstruction, internal accumulation of heat-toxin, and qi-yin deficiency	Bacterial toxins multiply in the bloodstream and spread systemically, causing toxic alterations in various tissues and organs. These alterations include pulmonary cellular turbidity, focal necrosis, and infiltration of inflammatory cells	Wang and Wang (2023), Xu Y. et al. (2024)

The current treatment landscape for ALI remains limited, with conventional therapies primarily comprising protective mechanical ventilation, glucocorticoid therapy, and fluid management. However, these approaches are often accompanied by significant side effects. For example, glucocorticoids can suppress the hypothalamic-pituitary-adrenal (HPA) axis, potentially leading to osteoporosis and other adverse effect (Schäcke et al., 2002). Similarly, mechanical ventilation, particularly when administered at high pressures or volumes, can aggravate ALI (Del Sorbo et al., 2011). In contrast, TCM offers a promising alternative, with a long-standing history in treating ALI, and has shown unique therapeutic benefits (Zhang et al., 2021; Jiang N. et al., 2021).

Recent years have seen an increase in research exploring the role of TCM in preventing and treating ALI. However, most existing reviews predate 2022 and do not incorporate the latest findings from the past 2 years (Liu Y. et al., 2024; Zhang, 2020). This paper aims to address this gap by providing a comprehensive review of significant advancements in ALI research over the past 5 years, with particular focus on recent discoveries related to its pathogenesis and molecular mechanism. Special attention is given to the pharmacological mechanisms and clinical application research of TCM in treating ALI since 2022, as well as the clinical efficacy of newly identified TCM formulations and their active components. By integrating the latest insights from both traditional Chinese and Western medicine, this review seeks to offer innovative strategies and perspectives for the holistic treatment of ALI.

## 2 Mechanisms of ALI pathogenesis

The pathogenesis of ALI is highly complex, involving a variety of physiological and pathological processes, including immune cell disfunction, alterations in cytokine profiles, disruption of the alveolar gas-blood barrier, organelle dysfunction, and ferroptosis. While classical mechanisms such as oxidative stress and inflammatory responses have been extensively discussed (Su et al., 2012), this review will briefly summarize these fundamental aspects while highlighting recent advancements and novel insights in ALI research over the past 5 years.

### 2.1 Dynamic alterations in immune cell composition and functionality

Dysregulation of the inflammatory response is a pivotal factor in ALI pathogenesis, encompassing not only aberrant expression of inflammatory factors but also intricate alterations in immune cell phenotypes and functions. Recent advances in cutting-edge technologies, such as single-cell sequencing, spatial transcriptomics, and spatial metabolomics, have enabled researchers to analyze the dynamic changes in cell types, migration patterns, and complex interaction networks within the lung microenvironment with unprecedented precision (Yu Y. et al., 2024; Wang Y. et al., 2024). These breakthroughs have significantly enhanced our understanding of ALI’s pathological mechanisms and provided novel directions for developing precision treatment strategies. This sections reviews recent progress on lung immune cell lineages in ALI, focusing on functional changes, underlying mechanisms, and their implications for disease progression.

#### 2.1.1 Alveolar macrophages

As the primary innate immune effector cells in the lung, alveolar macrophages play a critical role in ALI. Upon pathogen invasion, they promote the secretion of pro-inflammatory cytokines, which polarize neutrophils and mononuclear macrophages, and activating effector T cells. In the early stages of ALI, intratracheal administration of lipopolysaccharide (LPS) induces necrosis of alveolar macrophages and releases IL-1α, compromising the integrity of pulmonary endothelial cells (PEC) and facilitating neutrophil extravasation, thus exacerbating ALI progression (Dagvadorj et al., 2015). Macrophage polarization enhances the expression of pro-inflammatory cytokines (e.g., TNF-α, IL-6, IL-12) and chemokines (e.g., CCL8, IL-23), as well as oxidative stress factors like COX-2 and iNOS, all of which contribute to lung injury (Chen et al., 2020). Additionally, macrophages activate the NLRP3 inflammasome by upregulating key enzymes in the glycolysis signaling pathway, including HK1 and PKM2, which accelerate pyroptosis (Luo et al., 2022). Myeloid cells also express triggering receptor 1 (TREM-1), which reprograms macrophage metabolism, enhances glycolytic activity, activates the NLRP3 inflammasome, and triggers an inflammatory response in ALI (Zhong, 2023). The process of panoptosis in macrophages during ALI is regulated by the ZBP1 transcription factor. Knockdown of ZBP1 reduces key markers of inflammation and cell death (e.g., Caspase-3 p17/19, Caspase-1 p20, GSDMD p35, and Phospho-MLKL), thereby alleviating ALI in septic mice (Sun, 2024).

#### 2.1.2 Neutrophils

In ALI, capillary endothelial and epithelial cells sustain significant damage, while alveolar macrophages are activated to release cytokines (e.g., TNF-α, IL-1β) and chemokines, recruiting neutrophils into the lungs (Bhatia et al., 2012). Infiltrating neutrophils degranulate, releasing bactericidal proteins and pro-inflammatory cytokines, further exacerbating the inflammatory response (Aulakh, 2018). External stimuli can activate neutrophils, leading to the formation of neutrophil extracellular traps (NETs) and the release of reactive oxygen species (ROS) and proteases. This process activates the NLRP3 inflammasome, releasing inflammatory mediators and aggravating lung tissue injury (Li et al., 2018; Lin and Fessler, 2021; Lefrançais et al., 2018). In LPS-induced ALI models, Glycoprotein VI (GPVI) promotes neutrophil recruitment, platelet-neutrophil complexes and NETs, which subsequently trigger an inflammatory response (Burkard et al., 2023). The interaction between protein and fibrinogen in serum, via β-integrin on neutrophil surfaces, induces degranulation, abnormal neutrophil aggregation, and increased vascular permeability, contributing to ALI development (Soehnlein et al., 2008).

#### 2.1.3 T lymphocytes

T cells are pivotal in ALI pathogenesis. Regulatory T cells (Tregs) suppress the proliferation and differentiation of lung fibroblasts during LPS-induced lung inflammation (Tan et al., 2019; Seyran et al., 2023). Th17 cells, a subset of T helper cells, contribute significantly to host defense by secreting pro-inflammatory cytokines (e.g., IL-17A/F, IL-21, and IL-22) (Sakaguchi et al., 2016). At the molecular level, upregulation of IL-10 expression and inhibition of IL-35, RAGE, and Caspase-1 expression can attenuate T cells differentiation and mitigate ALI (Xie et al., 2021). In sepsis-induced ALI patients, elevated plasma nicotinamide phosphoribosyl transferase (NAMPT) levels induce T cells pyroptosis and immune dysfunction, which can be alleviated by the NAMPT inhibitor FK886 (Zheng, 2020).

#### 2.1.4 Other immune cells

Eosinophils, derived from bone marrow hematopoietic stem cells, play a role in immune regulation and allergic responses. Reduced eosinophil levels increase the risk of lung inflammation and mortality (Grisaru-Tal and Rothenberg, 2022). Conversely, IL-33-induced eosinophilia can mitigate the inflammatory response associated with *Staphylococcus aureus* lung infections (Krishack et al., 2021). Additionally, Natural killer (NK) lymphocytes can significantly alleviate inflammatory cell infiltration in lung immune injury and reduce the expression of IFN-γ expression in bronchoalveolar lavage fluid (Li et al., 2012).

### 2.2 Abnormal immune cytokines

#### 2.2.1 Interleukins and tumor necrosis factor

In ALI and ARDS, inflammatory mediators such as TNF-α, IL-1β, and IL-6 play a central role by mediating Cytidine monophosphate kinase 2 (CMPK2) (Chen D. S., 2022; Wang et al., 2021). IL-10 and IL-18 are critical in lung infections, compromising cell membrane integrity, stimulating alveolar neutrophils to produce chemokines, and promoting fibrosis via TGF-β and SMAD4 activation. This leads to production of pro-fibrotic proteins (e.g., ZO-1, MUC2) and fibroblast markers (e.g., FGF-1, αSMA) in epithelial cell, resulting in mesenchymal transformation and extracellular matrix collagen accumulation (Wang K. et al., 2022; Kandikattu et al., 2023). IL-33 induces neutrophil infiltration, increases alveolar endothelial barrier permeability and triggers alveolar epithelial cell death (Zou et al., 2023).

#### 2.2.2 Cell chemokines

Chemokines LCN2 and CCN3 regulate inflammatory responses in ALI. LCN2 enhances M2-type macrophage proportions via IL-6 and TNF-α (Zheng, 2023), while CCN3 promotes NF-κB p65, TGF-β1, TGF-βRⅡ, and p-Smad2/3 expression, increasing IL-6 and TNF-α production (Zhu et al., 2020). Knockdown of CCL2 reduces mononuclear macrophage recruitment and increases neutrophil expression, mitigating viral-induced lung injury (Lai et al., 2017). The CRTH2 receptor antagonist CT-133 improves macrophage and neutrophil infiltration, inhibits pulmonary vascular permeability, and suppresses inflammatory factor expression (Hussain et al., 2019).

#### 2.2.3 Other factors

Interferon (IFN) inhibits inflammatory responses to bacterial and viral infections and associated ALI (Verma et al., 2022). Inflammatory mediators activate the NLRP3 inflammasome in polymorphonuclear neutrophils (PMNs) and alveolar macrophages (AMs) (Cui et al., 2024). Heat shock protein HSP70 promotes lung inflammation in ALI mice but reduces CD36 receptor expression (Chen Y., 2022).

### 2.3 Impaired alveolar gas-blood barrier

In ALI, the alveolar-capillary barrier’s function is compromised, increasing capillary permeability and protein leakage into alveoli (Esquivel-Ruiz et al., 2021). Apoptosis plays a key role in this process; modulation of apoptotic genes (e.g., Caspase-3, Caspase-8) can enhance cell viability and mitigates inflammation (Yang et al., 2021; Ju et al., 2018; Li X. et al., 2019). The pulmonary intravascular glycocalyx, composed of hyaluronan (HA), heparan sulfate proteoglycan (HSPG), Syndecan-1, and Glypican-1, degrades during ALI, reducing lung surface protein expression (e.g., VE-Cadherin, Occludin, VCAM-1, E-selectin) (Chen et al., 2021; Cao et al., 2023). Alveolar epithelial cell apoptosis disrupts barrier integrity via the DAPK1 ligand pathway (Wang Y. et al., 2020). Cytoskeletal protein stability is crucial for barrier maintenance, facilitating intracellular signaling, tight junctions, and alveolar permeability (Yang et al., 2023). Inhibition of MMP-9 degradation, stabilization of endothelial cytoskeletal proteins, and Rho kinase pathway-mediated cytoskeletal remodeling have been shown to alleviate lung injury (Müller et al., 2019; Wu et al., 2016).

### 2.4 Mesenchymal stem cells (MSCs)

Mesenchymal stem cells (MSCs) have emerged as a promising therapeutic avenue for ALI due to their self-renewal capacity, multidirectional differentiation potential, and ability to modulate inflammation and fibrosis. Exosomes derived from bone marrow-derived MSCs (BM-MSCs) have been shown to inhibit several key inflammatory proteins, such as p65, IKKβ, p-IκBα, p-IκBβ, Caspase-1, GSDMD, and NLRP3, thereby suppressing macrophage pyroptosis (Xiao et al., 2020; Liu P. et al., 2024). Human placental MSCs (hPMSCs) protect the alveolar epithelial barrier through the ACE2/Ang (1–7) axis, downregulating TNF-α, IL-1, IL-6, and IL-10 levels, and alleviating endothelial injury (Yu W. et al., 2024; Xu et al., 2020). Human umbilical cord MSCs (hUC-MSCs) promote Trem2 expression, inhibit the NRF2/NF-κB/NLRP3 pathway, and reduce the release of TNF-α, IL-6, and IL-1β (Che et al., 2024). Overall, MSCs show considerable potential in the treatment of ALI, particularly in terms of protecting the alveolar barrier, modulating inflammatory responses, and exerting anti-fibrotic effects. Future research will aim to optimize MSC-based therapies, enhancing their efficacy and safety for clinical application.

### 2.5 Abnormal organelle function

#### 2.5.1 Endoplasmic reticulum stress (ERS)

Endoplasmic reticulum stress (ERS) has been recognized as a significant contributor to ALI pathogenesis (Mo and Hu, 2019). ERS can induce M1 macrophage polarization, disrupting the balance between M1 and M2 macrophages and driving excessive inflammation. Specifically, the IRE-1/XBP-1 signaling pathway activated by ERS, leads to lung epithelial cell death and NLRP3 inflammasome activation, thereby accelerating ALI progression (Zhao et al., 2020; Uddin and Barabutis, 2019).

#### 2.5.2 Abnormal mitochondrial function

Mitochondrial dysfunction plays a central role in ALI, with mitochondrial autophagy, dynamic imbalance, and metabolic dysfunction contributing to disease progression (Zong et al., 2024). This section focuses on mitochondrial autophagy and dynamic imbalance, while mitochondrial metabolic abnormalities are addressed in Section 2.7.

##### 2.5.2.1 Mitochondrial autophagy

Mitochondrial autophagy, or mitophagy, is essential for maintaining cellular homeostasis and preventing inflammation by removing damaged mitochondria. Disruption of mitophagy can lead to release of mitochondrial DNA (mtDNA), which activates Toll-like receptor 9 (TLR9), triggering inflammation. Elevated mitophagy, induced by deficiency in serine-activated protein kinase 3 (MMK3), enhances histone deacetylase activity and exacerbates lung injury (Tan and Chen, 2020). The PINK1/PARKIN pathway is pivotal in mitophagy regulation, where NLRP3 upregulates PINK1 expression, promoting mitophagy. However, inhibition of PINK1 abolishes this effect. PARKIN-mediated degradation of PARIS increases PGC-1α expression, promoting mitochondrial biogenesis and offering protection against hyperoxia-induced damage (Um and Yun, 2017).

##### 2.5.2.2 Mitochondrial dynamic imbalance

Imbalance between mitochondrial fusion and fission also contribute to ALI. In LPS-induced ALI models, fusion proteins Mfn1/2 and OPA1 are downregulated, while fission proteins Drp1 and Fis1 are upregulated, disrupting mitochondrial function and morphology (Shi et al., 2021). Additionally, activation of the Panx1 gene triggers the CAN-DRP1 signaling pathway, inducing mitochondrial dynamic imbalance, disrupting tight junctions, and promoting apoptosis, all of which contribute to ALI (Xu, 2023).

### 2.6 Iron death (ferroptosis)

Ferroptosis, an iron-dependent form of cell death characterized by lipid peroxidation, exacerbates ALI by causing oxidative damage to the alveolar membrane. In LPS-induced ALI models, elevated Fe^2+^ levels, decreased expression of ferroptosis markers (SLC7A11, GPX4), and increased lipid peroxidation (measured by propylene glycol) are observed in bronchial epithelial cells (Cai et al., 2022a). In sepsis-induced ALI, reduced GPX4 expression and increased MDA and Fe^2+^ levels further worsen lung injury (Cao et al., 2022; Long et al., 2020). Conversely, silencing mixed lineage kinase 3 (MLK3) mitigates LPS-induced epithelial damage by inhibiting p53-mediated ferroptosis (Liu et al., 2020). Furthermore, deficiency in the UTX/UTY protein family alleviates LPS-induced ALI by blocking ferroptosis in alveolar epithelia through NRF2 activation (Peng et al., 2021).

### 2.7 Metabolic disorders

Metabolic dysregulation plays a crucial role in ALI and ARDS, with altered sugar, lipid, amino acid, and glutathione metabolism being key contributors to disease progression (He et al., 2024).

#### 2.7.1 Sugar metabolism

During M1-type macrophage polarization, hexokinase (HK) transitions from the low-affinity isoform HK4 to the high-affinity isoform HK2, which exhibits enhanced catalytic activity (Bustamante and Pedersen, 1977). HK2 binds to mitochondrial voltage-dependent anion channels (VDAC), localizing to the outer mitochondrial membrane (Wang et al., 2014). Polyunsaturated fatty acids (PUFAs) inhibit HK2 binding to VDAC (Colquhoun, 2002), acting as anti-inflammatory agents that promote the conversion from the M1 to the M2 phenotype (Kawano et al., 2019). HK2 expression correlated with the SLC7A11/GPX4 axis, and HK2 deficiency results in increased macrophage death (Tan et al., 2022). PKM2, upregulated in M1 macrophages, regulates the Warburg effect by translocating to the nucleus and binding HIF-1α, promoting the inflammatory metabolic response that exacerbates lung injury (Yu et al., 2021; Christofk et al., 2008; Wang J. et al., 2022). Reducing PKM2 activity enhances the Warburg effect, further driving lung injury (Li et al., 2021). Nrf2 is a key regulator of glucose metabolism, and its knockdown reduces the expression of glycolytic genes, inhibiting the glycolytic pathway (Osburn et al., 2006; Zhang et al., 2018).

#### 2.7.2 Lipid metabolism

Phospholipid hydroperoxides, derived from PUFAs, are key products of lipid peroxidation and play a significant role in ALI. Fatty acyl-CoA synthase long-chain family member 4 (ACSL4) participates in lipid biosynthesis and catalyzes the conversion of PUFAs to PUFA-PLS, compromising plasma membrane integrity (Wang Y. et al., 2022). HIF-1α binds to the ACSL4 promoter and negatively regulates its transcription, while activating Nrf2 to enhance glycolysis (Li et al., 2024). Some hydroperoxides, along with free PUFAs, act as substrates for lipid signaling. The esterification of PUFAs to acyl-CoA by long-chain acyl-CoA synthetase and their subsequent incorporation into membrane phospholipids contributed to ALI pathogenesis by activating toll-like receptors, thereby exacerbating inflammation (Zou et al., 2019; Imai et al., 2008).

#### 2.7.3 Amino acid and glutathione metabolism

Glutathione (GSH), an important antioxidant, plays a crucial role in reducing reactive oxygen species (ROS) and preventing oxidative damage (Xie et al., 2016). GSH synthesis depends on the cystine/glutamate antiporter system, which exchanges extracellular cystine for intracellular glutamate, a precursor for GSH synthesis. Under the catalytic activity of GPX4, GSH reduces lipid hydroperoxides to non-toxic alcohols, with its depletion leading to increased oxidative stress and cell death (Yang et al., 2014). Fatty acid-binding proteins derived from glutathione can mitigate ALI by modulating inflammatory responses and inhibiting Grx1 expression (Guo Y. et al., 2021).

#### 2.7.4 Mitochondrial metabolism disorder

Mitochondria are central to cellular energy metabolism, and their dysfunction contributes significantly to ALI. Pathological mitochondrial changes, such as shrinkage, altered membrane density, and compromised membrane integrity, are common in ALI. Inhibition of Mucin-1 dimerization disrupts mitochondrial function by downregulation GPX4, GSH and SOD expression, leading to lipid peroxide accumulation and mitochondrial damage. Targeted mitochondrial antioxidants, such as MitoQ, can reduce mitochondrial ROS, inhibit ferroptosis, and alleviate ALI (Kufe, 2020; Wang Y. M. et al., 2022; Zhan et al., 2022; Bock and Tait, 2020).

#### 2.7.5 Other regulatory mechanisms

Exosome, small vesicles containing RNA and proteins, serve as important mediators of intracellular communication, immune responses, and cellular regulation (Wang and Zhu, 2019; Jiang et al., 2019; Tavasolian et al., 2021). In ALI, exosomes derived from alveolar macrophages have been shown to regulate the Hippo signaling pathway via tRNA-derived fragments, participating in LPS-induced ALI (Wang W. et al., 2022).

### 2.8 Intestinal bacteria factor

The intestinal microbiota, comprising bacteria like *Escherichia coli* and *Lactobacillus*, plays a critical role in immune modulation (Zhou et al., 2020). Disruption of the gut microbiota can lead to systemic inflammation, contributing to ALI. Research suggests that the TLR-endoplasmic reticulum stress-ROS signaling pathway, activated by intestinal barrier dysfunction with gut microbiota, exacerbates lung injury by promoting systemic inflammatory responses (Gong et al., 2022).

## 3 Study on mechanism of TCM treatment of ALI

### 3.1 Compounds

Recent studies on the use of TCM for ALI have largely focused on therapies aimed at clearing heat and detoxifying, regulating fu-organs, and resolving blood stasis. Over the past 3 years, a significant body of clinical research has emerged in investigating TCM compound treatments for ALI (Gasmi et al., 2024; Ren, 2020; Li and Liang, 2019; Guo J. et al., 2021). These studies have helped to deepen our understanding of the mechanisms through which TCM influences ALI, establishing a foundation for evaluating the clinical efficacy of TCM-based therapies and supporting the development of novel therapeutic agents in the future.

#### 3.1.1 Heat clearance and detoxification

The development of ALI is closely linked to the accumulation of pathogenic heat, which invades the lungs, cause fluid injury, depletes qi, and obstructs lung qi. Therefore, heat-clearing prescriptions are a key therapeutic approach in TCM for treating ALI (Lu et al., 2020). Studies have demonstrated that TCM formulas like Shenlian and Retong Fang markedly reduced mRNA levels of inflammatory factors (IL-6, IL-18 and MCP-1) and decrease protein levels of CD68, SP-A, and CC16 in rat lung tissue with ALI (Yang et al., 2022; Zhang et al., 2010).

The Wenqing Decoction has been found to inhibit the PI3K/AKT signaling pathway, reducing levels of TNF-α, IL-6, IL-1β, RAGE, and various phosphorylated proteins (PI3K, AKT, p-PI3K, p-AKT) (Xie et al., 2024). Chaihuqingwen and Zukamu Granules have also proven effective by inhibiting phosphorylation of NLRP3, TLR4, NF-κBp65, and IκBα in rat lung tissue, along with caspase-1 mRNA and protein expression, thus modulating inflammatory responses (Zhou et al., 2023; Yu C. et al., 2024).

The Qingfeilitan Formula reduces mRNA levels of TNF-α, IL-6, IL-1β, and MDA in alveolar lavage fluid, while increasing the activity of antioxidants like SOD and GSH-Px, providing anti-inflammatory and antioxidant effects (Diao et al., 2022). Similarly, Qingfei Paidu Decoction inhibits macrophage polarization, downregulates IL-6, TNF-α, MIP-2, MCP-1, upregulates IL-10, and reduces nuclear translocation of key inflammatory mediators (TAK1, IKK, and p65) (Ye et al., 2023).

Additionally, some formulas like Yindan Jiedu Capsule, Xuanfei Baidu Decoction and Tanreqing target various inflammatory pathways, including the MAPK/NF-κB and NF-κB signaling pathways, offering broad anti-inflammatory effects (Feng et al., 2022; Li et al., 2023; Hu et al., 2022). Shufengjiedu Capsule prevents macrophage apoptosis by increasing A2A adenosine receptor expression and inhibiting NF-κB phosphorylation (Cai et al., 2022b). Maxingshigan Decoction has been found to reduce lung inflammation, enhance antioxidant activity, and regulate key inflammatory pathways including MAPK/NF-κB pathway (Hou et al., 2023).

Qingjie Huagong Formula has shown promise in improving lung and pancreatic and lung tissue pathology in SAP-ALI rat models, inhibiting the PI3K/AKT1 signaling pathway (Feng et al., 2024). Gegen Qinlian Decoction has been found to reduce lung inflammation, enhance antioxidant activity, and suppress C3, C5a, and IL-17 expression (Li W. et al., 2022).

#### 3.1.2 The catharsis category of promoting bowel clearance and pulmonary Detoxification (Tongfu)

The Tongfu approach, known for its cathartic properties, is crucial in managing ALI. Tongfu Formula, in particular, inhibits the expression of pro-inflammatory cytokines (IL-6, IL-1β, IL-18), STING pathway components (p-STING, STING) and inflammasome proteins (NLRP3, ASC) in mice with sepsis-induced ALI (Huo and Yue, 2024). Similarly, Tongfu Xiefei Enema Solution reduces mRNA expression of TNF-α, IL-1β, and p38 MAPK, as well as the protein expression of p38, p-MLC/MLC2, and MLCK, contributing to improved ALI outcomes (Ma et al., 2024).

Tingli Dazao Xiefei Decoction and Xuanbai Chengqi Decoction demonstrate efficacy in reducing inflammation by regulating the PI3K, mTOR, HIF-1α, and p-AKT/AKT pathways, while also inhibiting glycolysis in lung tissue (Zhang et al., 2023; Wang S. et al., 2024; Zhu et al., 2021). Research into lung-intestinal co-treatment for ALI has shown that the Dachengqi Decoction alleviates inflammation by regulating the NF-κB/NLRP3 signaling pathway (Kou et al., 2022).

#### 3.1.3 Enhancing blood circulation and resolving blood stasis

TCM formulas aimed at enhancing blood circulation and resolving blood stasis, such as Huoxue Huayu Decoction, play a significant role in treating ALI by activating qi and promoting blood flow. These formulas help mitigate lung damage and enhance the expression of tight junction proteins (ZO-1, Occludin, AQP-1, AQP-5) in the lung gas-blood barrier (Qin et al., 2025).

Qidong Huoxue Yin inhibits apoptosis in lung epithelial cells and reduces inflammatory cytokines like TNF-α, IL-6, and chemokines in mice with ALI (Zheng et al., 2021). Xuebijing Injection mitigates inflammatory infiltration in ALI by antagonizing neutrophils and other immune cells (Zheng and Zhang, 2024). Other formulas, such as Qilongtian Capsule and Mailuo Yin regulate the NF-κB pathway and suppress inflammation by reducing the phosphorylation of MyD88, IκBα, and NF-κB (Luo et al., 2024; Miao et al., 2022).

#### 3.1.4 Other formulas

Formulas like Xiaoxuming Decoction, traditionally used for stroke treatment, have shown potential in alleviating lung inflammation associated with ALI (Pei et al., 2023). Research indicates that Xiaoxuming decoction reduces levels of TNF-α, IL-1β and IL-8 in LPS-induced cells and mitigates pyroptosis by inhibiting key inflammasome proteins (USP9X, NLRP3, IL-1β, Caspase-1) (Xiang et al., 2022; Xiang et al., 2021).

**FIGURE 1:**
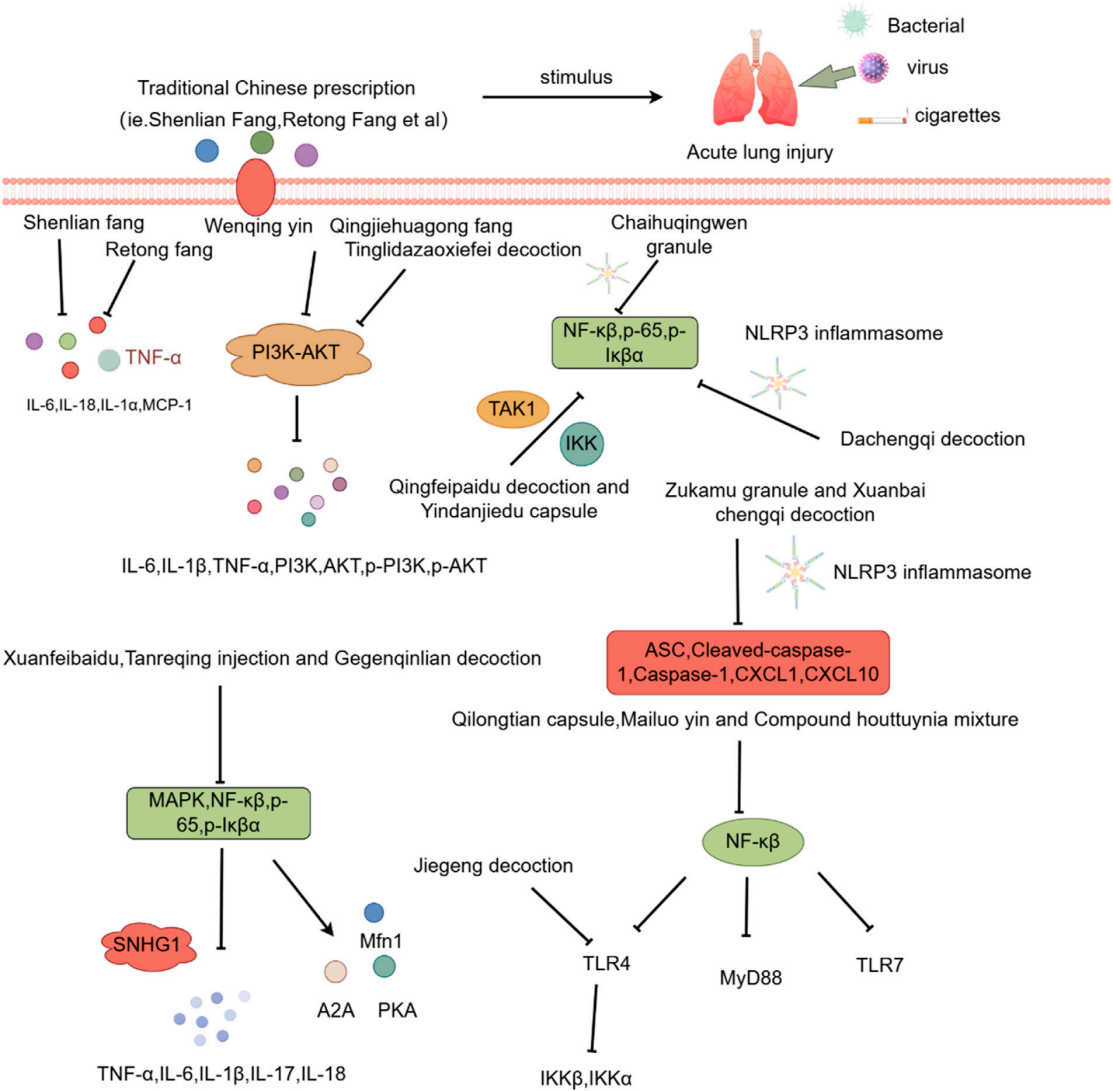
Investigation into the mechanism of TCM Compound in treating ALI.

Similarly, both Jiegeng Decoction and Compound Houttuynia mixture reduce oxidative stress and inflammatory responses by inhibiting ROS production, mitochondrial membrane potential polarization, and the expression of inflammatory mediators, apoptotic proteins (Cleaved Caspase 3, Bax, Bcl2) and pyrogenic proteins (p-NF-κB,NLRP3, ASC, Cleaved-Caspase 1 and Cleaved-GSDMD) (Li, 2023; Wei, 2024). Further details are provided in Table 2, Figure 1.

**TABLE 2:** Summary of TCM Compounds for the prevention and treatment of ALI.

Principles of TCM	Herb/Compound	Signal pathway	Dose	Index detection	Constitution
Heat Clearance and Detoxification	Shenlian Fang	/	8.64 g/kg	IL-6, IL-18, IL-1α, MCP-1, SP-A, CC16↓	*Panax ginseng* C. A. Mey., *Coptis chinensis* Franch., *Setaria italica* (L.) Beauv., *Citrus reticulata* Blanco, *Nelumbo nucifera* Gaertn., *Poria cocos* (Schw.)Wolf, et al.
	Retong Fang	/	1 g/kg	TNF-α, IL-6, IL-18, IL-1α↓	*Arnebia euchroma* (Royle) Johnst., *Eschenbachia blinii* (H.Lév.) Brouillet, *Gentiana macrophylla* Pall., et al.
	Wenqing Yin	PI3K/AKT	10.8 g/kg	TNF-α, IL-6, IL-1β, RAGE, PI3K, p-PI3K, AKT, p-AKT↓	*Angelica sinensis* (Oliv.)Diels, *Paeonia lactiflora* Pall., *Ligusticum chuanxiong* Hort., *Coptis chinensis* Franch., et al.
	Chaihuqingwen Granule	TLR4/NF-κB/NLRP3	8 g/kg	TLR4, NLRP3, NF-κB p65, p-IκBα↓	*Bupleurum chinense* DC., *Astragalus membranaceus* (Fisch.)Bge., *Lonicera japonica* Thunb., *Coix lacryma-jobi L.var.mayuen* (Roman.) Stapf, et al.
	Zukamu Granule	NLRP3/Caspase-1/GSDMD	2.34 g/kg	NLRP3, ASC, Caspase-1, Cleaved-caspase-1 p20, IL-1β, IL-18↓	*Rheum palmatum* L., *Glycyrrhiza uralensis* Fisch., *Mentha haplocalyx* Briq., et al.
	Qingfeilitan Formula	TNF-α,Toll-Like	10 mg/mL	TNF-α, IL-6, IL-1β↓; SOD, GSH-Px↑	*Gypsum* Fibrosum, *Scutellaria baicalensis* Georgi, *Trichosanthes kirilowii* Maxim., et al.
	Qingfei Paidu Decoction	TAK1/IKK/NF-κB	/	IL-6, TNF-α, MIP-2, MCP-1, TAK1, IKK p-p65↓; IL-10↑	*Ephedra sinica* Stapf, *Prunus armeniaca* L.var.ansu Maxim., *Poria cocos* (Schw.)Wolf, *Bupleurum chinense* DC., et al.
	Yindan Jiedu Capsule	NF-κB	30% medicated serum	IκBα/p-IκBα, p65, TNF-α, IL-6, IL-1β, NO↓	*Morus alba* L., *Scutellaria baicalensis* Georgi, *Descurainia Sophia* (L.)Webb. ex Prantl., *Lonicera japonica* Thunb., *Scrophularia ningpoensis* Hemsl., et al.
	Xuanfei baidu Decoction	MAPK/NF-κB	0.5 mg/mL; 2.16 g/kg	p-IKKα, p-NF-κB, p-IκBα, p-p38, p-Erk, TNF-α, IL-6, IL-1β, PGC-1α, LC3B, Nrf1, JC-1, NLRP1, NLRP3, Caspase11↓; Mfn1, Mfn2, ATP↑	*Ephedra sinica* Stapf, *Prunus armeniaca L.var.ansu* Maxim., *Gypsum* Fibrosum, *Coix lacryma-jobi L.var.mayuen* (Roman.)Stapf, *Atractylodes lancea* (Thunb.)DC., et al.
	Tanreqing	HMGB1/NF-κB/SNHG1	4 mL/kg	p-p65, p-p38, HMGB1, SNHG1, TNF-α, IL-6, IL-1β, IL-18, MCP-1, ROS↓	*Scutellaria baicalensis* *Georgi*, *Selenarctos thibetanus* (*G. Cuvier*), *Capra hircus* L
	Shufeng Jiedu Capsule	A2A/NF-κB	0.5 mg/mL	A2A, p-IκB/IκB, p-p65/p65, Cc3, Bax/Bcl-2↓; PKA↑	*Polygonum cuspidatum Sieb. et* Zucc*., Forsythia suspensa* (Thunb.)Vahl*, Isatis indigotica Fort., Bupleurum chinense.*DC*, Dahurian* Patrinia*, Verbena officinalis* L.*,Phragmites communis* Trin.*, Glycyrrhiza uralensis* Fisch
	Maxingshigan Decoction	MAPK/NF-κB	5.4 g/kg	IL-1β, IL-6, TNF-α, p38MAPK, JNK, ERK1/2↓; MDA, SOD, GSH↑	*Prunus armeniaca L.var.ansu* Maxim., *Gypsum* Fibrosum, *Polygonum cuspidatum* Sieb. et Zucc., *Glycyrrhiza uralensis* Fisch
	Qingjie Huagong Formula	PI3K/AKT1	4.5 g/kg	TNF-α, IL-1β, IL-6↓	*Bupleurum chinense* DC., *Scutellaria baicalensis* Georgi, *Salvia miltiorrhiza* Bge., et al.
	Gegenqinlian Decoction	MAPK/NF-κB	1.25 g/kg	TNF-α, IL-6, IL-1β, C3, C5a,IL-17↓	*Pueraria lobata* (Willd.)Ohwi, *Coptis chinensis* Franch., *Glycyrrhiza uralensis* Fisch., et al.
The Catharsis Category of Promoting Bowel Clearance and Pulmonary Detoxification (Tongfu)	Tongfu Formula	mtDNA-STING-NLRP3	9.9 g/kg	IL-6, IL-1β, IL-18, p-STING, STING, NLRP3, ASC mRNA and protein ↓	*Rheum palmatum* L., *Prunus persica* (L.) Batsch, *Paeonia lactiflora* Pall., et al.
	Tongfu Xiefei Enema Solution	p38 MAPK/MLCK	7.37 g/kg	TNF-1α, IL-1β, p38 MAPK mRNA and p38, p-MLC/MLC2 MLCK protein↓	*Ephedra sinica* Stapf, *Prunus armeniaca L.var.ansu* Maxim., *Trichosanthes kirilowii* Maxim., et al.
	Tingli dazao xiefei Decoction	PTEN/PI3K/AKT	5.85 g/kg	IL-6, TNF-α, STAT3, SOC3, PI3K, AKT, p-AKT, PTEN↓	*Descurainia Sophia* (L.) *Webb.*ex, Prantl., *Ziziphus jujuba* Mill
	Xuanbai Cheng Qi Decoction	NLRP3 inflammasome	9 g/kg	NLRP3, Cleaved-Caspase1, GSDMD-N, IL-1β, pro-IL-1β, CXCL1, CXCL10, TNF-α, NLRP3, NF-κB P65 mRNA↓	*Gypsum* Fibrosum, *Rheum palmatum* L.,*Prunus armeniaca L.var.ansu* Maxim., et al.
	Dachengqi Decoction	NF-κB/NLRP3	5 g/kg	p-p65/p65, p-IκBα/IκBα, NLRP3, TNF-α, IL-6, IL-1β↓	*Rheum palmatum* L., *Magnolia officinalis Rehd.et* Wils., *Citrus aurantium* L., et al.
Enhancing blood circulation and resolving blood stasis	Huoxue Huayu Decoction	ZO-1/Occludin	7.2 g/kg	ZO-1, Occludin, AQP-1, AQP-5↑	*A.membranaceus* (Fisch.)Bge., *Codonopsis pilosula* (Franch.)Nannf., *Angelica sinensis* (Oliv.)Diels., et al.
	Qidong Huoxue Yin	iRhom2/TACE	8 mL/kg	iRhom2, TACE, TNF-α, IL-6↓	*A.membranaceus* (Fisch.)Bge., *Angelica sinensis* (Oliv.)Diels, *Rheum palmatum* L., et al.
	Xuebijing Injection	FPRs/NLRP3	12 mL/kg	FPR1, FPR2, NLRP3, IL-1β, IL-6, TNF-α↓	*Carthamus tinctorius* L., *Paeonia lactiflora* Pall., *Ligusticum chuanxiong* Hort., *Angelica sinensis* (Oliv.)Diels., et al.
	Qilongtian Capsule	TLR4/NF-κB	0.35 g/kg	TNF-α, IL-6, IL-1β, TLR4, NF-κBP65, NLRP3, MyD88 IκBα, NF-κBP65↓; IκBα↑	*Rhodiola crenulata* (Hook. f. et Thoms.) H. Ohba, *Panax notoginseng* (Burk.)F. H.Chen, *Pheretima aspergillum* (E.Perrier)
	Mailuo Yin	TLR4/NF-κB	1.25 μL/mL; 7.5 mL/kg	TLR4, MyD88, p-p65, p65, p-IκBα, TNF-α, IL-6, IL-1β, MPO↓	*Lonicera japonica* Thunb., *Achyranthes bidentata* BL., *Scrophularia ningpoensis* Hemsl., *Dendrobium nobile* Lindl
Others	Xiaoxuming Decoction	USP9X/NLRP3/Caspase-1/Pro Caspase-1	10 mg/L	USP9X, NLRP3, IL-1β, Pro-IL-1β, Caspase-1, Pro-Caspase-1↓	*Ephedra sinica* Stapf, *Panax ginseng* C. A.Mey., *Scutellaria baicalensis* Georgi., et al.
	Jiegeng Decoction	/	250 mg/kg	TLR4, p-IKKβ, p-IKKα and p-IκBα↓	*Platycodon grandiflorum* (Jacq.) A.DC., *Glycyrrhiza uralensis* Fisch
	Compound Houttuynia mixture	TLR7/MyD88/NF-κB	5.46 g/kg; 87.4 mg/mL	TRAF6, IRF7, MyD88↓	*Houttuynia cordata* Thunb., *Scutellaria baicalensis* Georgi, *Isatis indigotica* Fort., et al.

### 3.2 Monomer components

This section systematically investigates the effects of monomer components with clinical therapeutic potential for treating ALI. These monomers have demonstrated diverse mechanisms of action, suggesting their promise as effective treatments for ALI.

#### 3.2.1 Tanshinone ⅡA

Tanshinone ⅡA, when combined with carvedilol and simvastatin, is commonly used to treat cardiovascular diseases such as coronary heart disease and heart failure (Shao et al., 2022). Recent research has shown that tanshinone ⅡA can mitigate ALI by inhibiting key signaling molecules, including ROCK2, NF-κB, Pro-caspase, P65, and IκBα, all of which play roles in inflammation and cell death (Liu J. et al., 2022).

#### 3.2.2 Artemisinin

Known for its broad therapeutic potential against conditions like tumors, rheumatoid arthritis, and skin inflammation (Zhao et al., 2024; Liu Y. J. et al., 2022), artemisinin has also shown promise in ALI treatment. Studies suggest it alleviates lung injury by suppressing inflammatory factors, myeloperoxidase (MPO), malondialdehyde (MDA), and other oxidative stress markers. Additionally, it reduces the expression of p-AKT and HO-1 proteins, contributing to its protective effects on the lung injury (Ji et al., 2023).

#### 3.2.3 Glycyrrhizin

Glycyrrhizin, and its derivatives, have been shown to reduce serum inflammatory markers and transaminase levels in viral hepatitis (Guo, 2024). In ALI, glycyrrhizin helps by inhibiting ROS production in LPS-induced cells, downregulating p-PI3K and p-AKT protein levels, and reducing the formation of NLRP3 inflammasome. These actions help protect against ALI by mitigating oxidative stress and inflammation (Wang K. et al., 2020).

#### 3.2.4 Andrographolide

Primarily used to treat bacterial pneumonia and other inflammatory conditions (Xu, 2016; Song, 2022), andrographolide has been shown to inhibit NLRP3 inflammasome activation, reducing inflammatory factor and alleviating lung epithelial cell damage (Qin et al., 2024).

#### 3.2.5 Huperzine-A

Typically used in the treatment of neurodegenerative diseases like Alzheimer’s disease and vascular dementia (Wang et al., 2023; Lu et al., 2021), huperzine-A also exhibits nti-inflammatory effects in ALI. It reduces levels of IL-6, MDA, and ROS, while increasing SOD levels, which contribute to its therapeutic effects in lung injury (Shi et al., 2024).

#### 3.2.6 Cepharanthine

Although primarily used to treat leukopenia in cancer patients undergoing chemotherapy, cepharanthine has shown potential in treating ALI by improving immune function and reducing inflammation (Ge et al., 2023). Stephanolin, a related compound, has been shown to reduce viral RNA and virus production, suggesting its potential in viral-induced ALI (Fan et al., 2020).

#### 3.2.7 Chloroquine

As a 4-aminoquinoline antimalarial drug, chloroquine is alsoused to treat autoimmune diseases like rheumatoid arthritis. It works by improving lysosomal pH, inhibiting autophagosome-lysosome fusion, and enhancing CXCR4 expression, thereby reducing inflammation and alleviating ALI (Yan et al., 2013).

#### 3.2.8 Total paeony glycosides

These compounds inhibit mitochondrial ROS (mtROS) oxidative stress and block NLRP3 inflammasome activation. This significantly contributes to the treatment of ALI by reducing inflammation and cellular damage (Xu Y. J. et al., 2024).

#### 3.2.9 Diacerein

**FIGURE 2:**
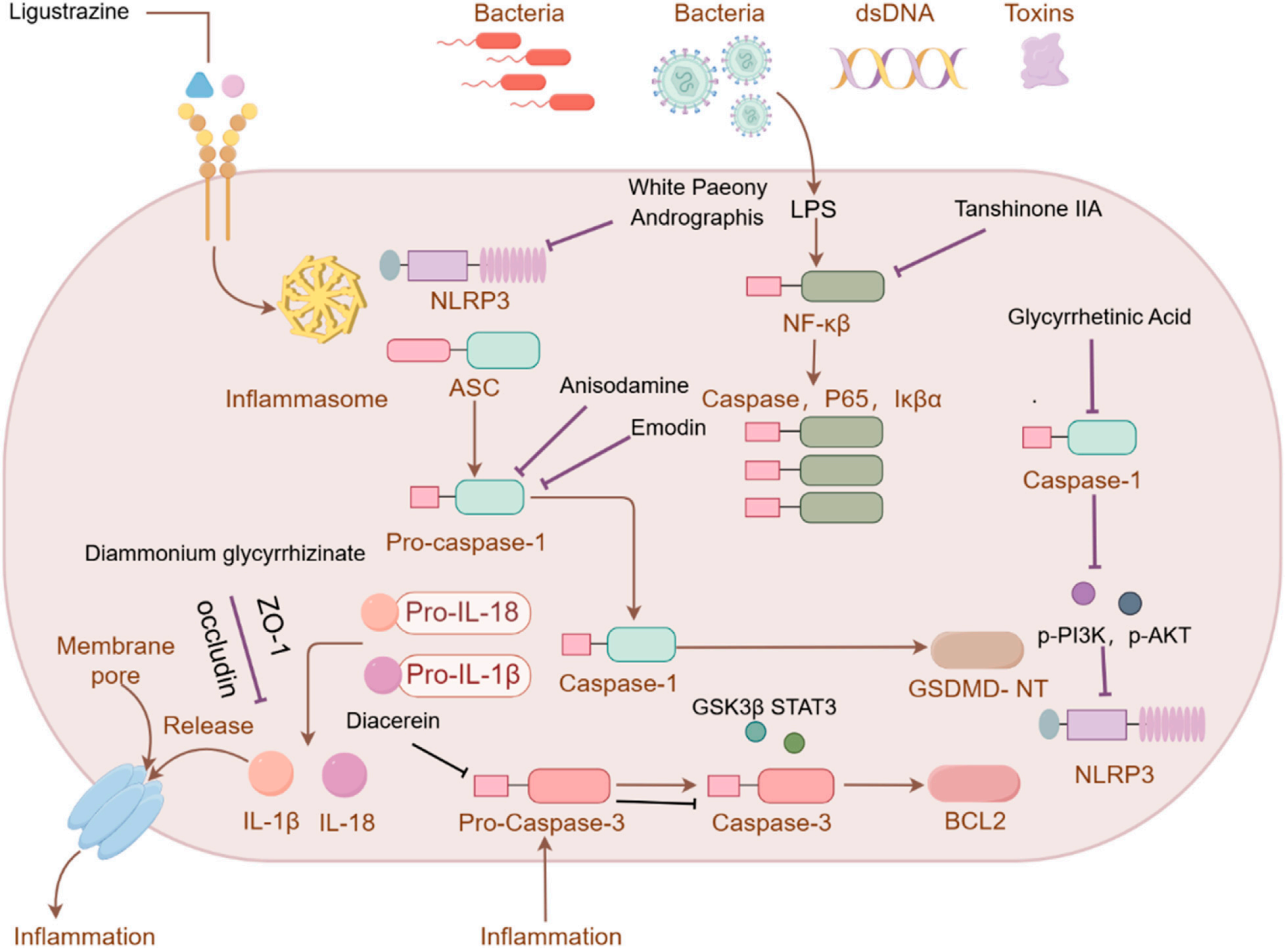
Investigation into the targets of TCM monomers and derivatives for the treatment of ALI.

As a derivative of rhein, diacerein is commonly used for degenerative joint diseases. Recently, diacerein has demonstrated potential in treating ALI by reducing the Sphk1/S1P axis and inhibiting GSK-3β, STAT3, and Caspase-3. It also increases BCL-2 protein expression, which plays a role in cell survival and inflammation regulation (Youssef et al., 2022). Further details are provided in Table 3, Figure 2.

**TABLE 3:** Investigation into the mechanism of monomer components in the treatment of ALI.

Monomer compounds	CAS	Molecular formula	*In vivo* and *in vitro* models	Pertinent pharmacological targets	Investigation of the relevant mechanism
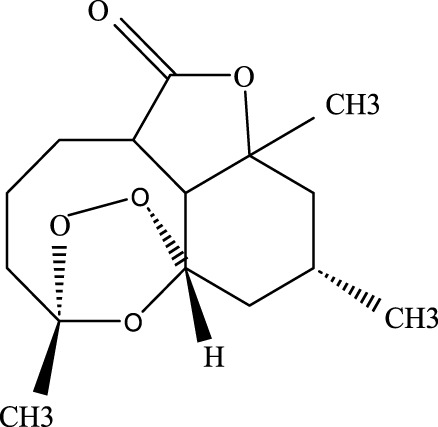 Artemisinin	63968–64-9	C_15_H_22_O_5_	Ischemia-reperfusion modeling	TNF-a, IL-6, IL-1β, MDA, MPO, p-AKT, HO-1↓	Artemisinin has the potential to suppress the expression of inflammatory responses, thereby mitigating ALI.
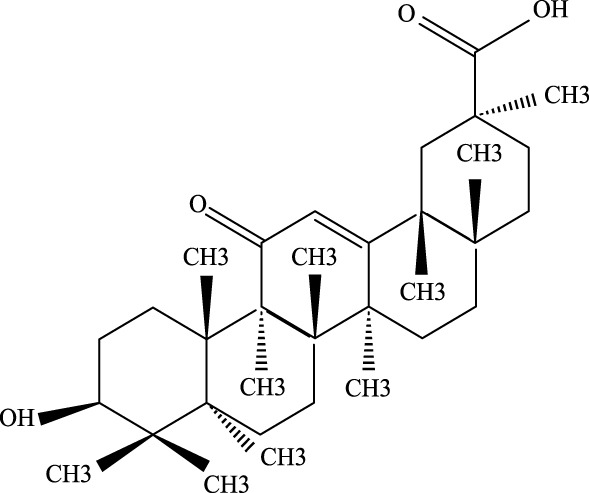 Glycyrrhetinic Acid	471–53-4	C_30_H_46_O_4_	Intraperitoneal administration of LPS at a dose of 10 mg/kg	ROS, p-PI3K, p-AKT, Cleaved Caspase-1 and NLRP3 ↓	Glycyrrhizic acid and its derivatives mitigate the production of reactive oxygen species (ROS) in LPS-induced cells, downregulate the protein levels of phosphorylated phosphatidylinositol 3-kinase (p-PI3K) and phosphorylated AKT (p-AKT), reduce the expression of cleaved Caspase-1 and NLRP3, and inhibit the formation of NLRP3 inflammasomes, thereby providing protection against ALI.
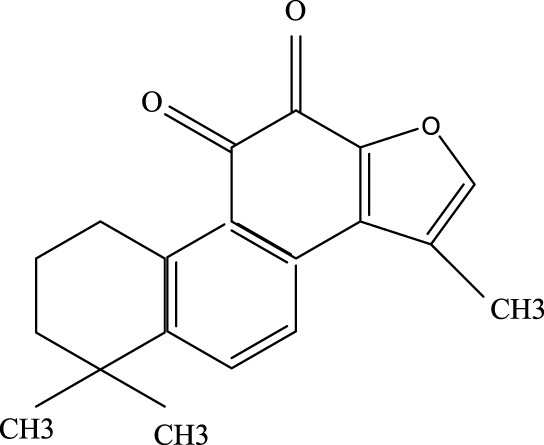 Tanshinone IIA	568–72-9	C_19_H_18_O_3_	LPS was used to induce RLE-6TN cells. The sepsis model was established through cecal ligation and puncture (CLP)	ROCK2, TNF-α, IL-1β, IL-6, Caspase, pro-caspase, P65, IkBα↓	TIIA improved sepsis-induced lung injury by downregulating ROCK2 and further inactivating the NF-κB signaling pathway *in vivo* and *in vitro*
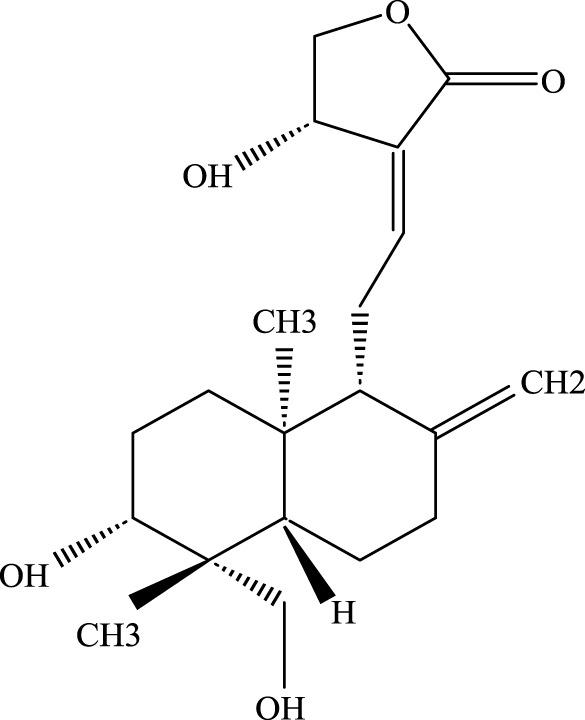 Andrographis	5508–58-7	C_20_H_30_O_5_	CLP mouse model	TNF-α, IL-1β↓	Andrographis suppresses the activation and release of inflammasomes, consequently inhibiting the secretion of inflammatory cytokines, which is beneficial for the treatment of ALI.
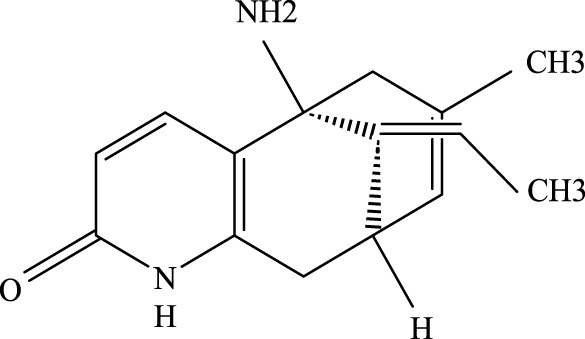 Huperzine A	102518–79-6	C_15_H_18_NO_2_	Intratracheal administration of LPS at a dose of 5 mg/kg	IL-6, MDA, ROS↓; SOD↑	Huperzine A can decrease the concentrations of IL-6, MDA, and ROS while increasing the concentration of SOD, thereby contributing to the treatment of ALI.
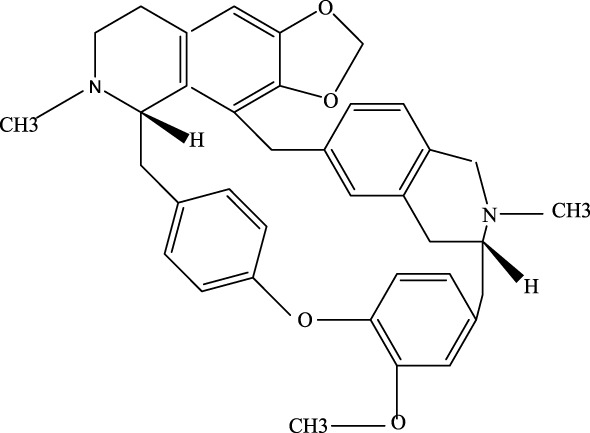 Cepharanthine	481–49-2	C_37_H_38_N_2_O_6_	/	/	Cepharanthine tablets significantly decrease viral RNA levels and inhibit viral replication, thereby mitigating acute lung injury
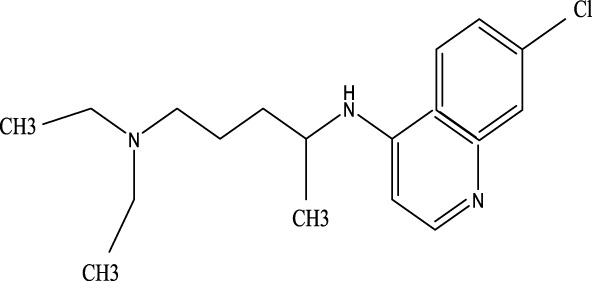 Chloroquine	54–05-7	C_18_H_26_ClN_3_	Infection with the H5N1 influenza virus	PH, CXCR4↑	Chloroquine elevates lysosomal pH, inhibits the fusion between autophagosomes and lysosomes, degrades lysosomal proteins, upregulates the expression of CXCR4 *in vivo*, thereby alleviating ALI.
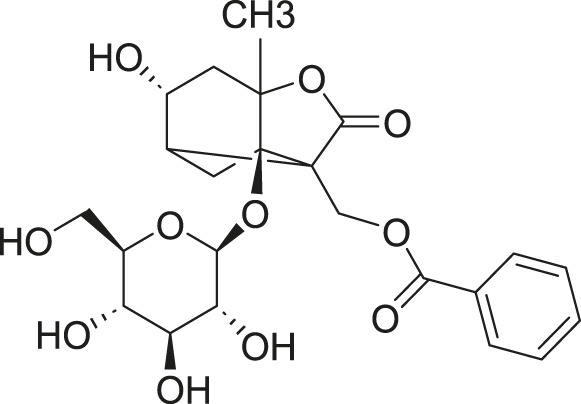 White Paeony	39011–90-0	C_23_H_28_O_11_	Intratracheal administration of LPS at a dose of 50 ng/mL	IL-1β, IL-18, IL-6, TNF-α, ASC, NLRP3↓	Total paeony glycosides capsules may ameliorate ALI in mice through the inhibition of NLRP3 inflammasome activation
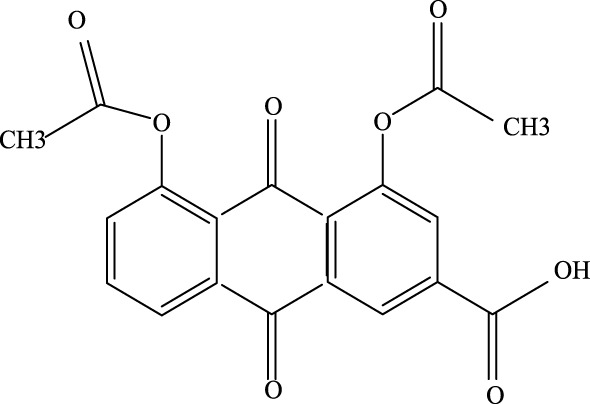 Diacerein	13739–02-1	C_19_H_12_O_8_	Intraperitoneal injection of LPS at 5 mg/kg	Sphk1/S1P, GSK-3β, STAT3, Caspase-3↓; BCL-2↑	Diacerein can modulate the Sphk1/S1P axis, reduce the levels of GSK-3β, STAT3, and Caspase-3, while increasing the expression of BCL-2 protein, thereby contributing to the treatment of acute lung injury

## 4 Clinical research on the treatment of acute lung injury with Traditional Chinese Medicine

### 4.1 The significance and current status of clinical research

The application progress of traditional Chinese medicine (TCM) in the clinical treatment of acute lung injury (ALI) is substantial, demonstrating promising therapeutic potential. As research advances from cell culture to clinical trials, findings from basic studies are increasingly being translated into clinical practice. Randomized controlled trials (RCTs) serve as a critical methodology for evaluating the efficacy of TCM interventions in ALI. These trials provide valuable evidence and a scientific foundation for understanding the potential benefits of TCM in managing ALI (Zheng et al., 2024).

In recent years, a growing number of high-quality clinical studies have demonstrated the notable efficacy of traditional Chinese medicine (TCM) in treating acute lung injury (ALI). Particularly within an integrated treatment framework combining both TCM and Western medicine, this approach has been shown to effectively alleviate clinical symptoms, modulate inflammatory responses, improve oxygenation function, and significantly reduce patient mortality rates.

### 4.2 Clinical evidence of integrated traditional Chinese and Western medicine in treatment

#### 4.2.1 Clinical application of compound preparations

##### 4.2.1.1 Baofeijiejiong mixture

A randomized controlled study involving 60 patients with acute lung injury (ALI) caused by sepsis demonstrated that the integration of traditional Chinese and Western medicine treatments—specifically Pulmonary Protective and Baofeijiejiong mixture—significantly reduced the 7-day mortality rate among ALI patients compared to treatment with Western medicine alone. Furthermore, this integrative approach enhanced the overall efficacy of Western medical treatment. Following the intervention, notable improvements were observed in traditional Chinese medicine (TCM) symptom scores, including symptoms such as fever, dyspnea, and sputum production. Inflammatory markers, such as IL-8 and TNF-α, showed a marked decrease, while organ function indicators, including SOFA and APACHE II scores, significantly improved. Additionally, levels of biomarkers such as white blood cell count, C-reactive protein (CRP), and procalcitonin (PCT) were also reduced. Importantly, this therapeutic strategy decreased the need for invasive mechanical ventilation, thereby lowering the incidence of acute respiratory distress syndrome (ARDS) and multiple organ dysfunction syndrome (MODS) in patients with sepsis-induced ALI (Su, 2015).

##### 4.2.1.2 Dachengqi Decoction

In a clinical study involving 68 patients with acute lung injury (ALI), administration of Dachengqi Decoction via nasogastric feeding demonstrated significant efficacy in reducing the Traditional Chinese Medicine (TCM) syndrome score of Yangming Fu in postoperative ALI patients, thereby effectively alleviating clinical symptoms such as profuse sweating, abdominal distension, and pain. Laboratory analyses revealed that following treatment, levels of C-reactive protein (CRP), interleukin-1 (IL-1) and interleukin-6 (IL-6) were markedly decreased, while the alveolar-arterial oxygen pressure difference P (Aa)o_2_ improved significantly. Additionally, the duration of mechanical ventilation was notably shortened (Li Y. et al., 2022).

##### 4.2.1.3 Yiqihuoxuexiezhuo formula

Professor Chao Enxiang, a master of traditional Chinese medicine, formulated a therapeutic regimen aimed at tonifying qi, resolving turbidity, and promoting blood circulation. This formula demonstrated significant efficacy in a randomized controlled trial involving 60 patients with acute lung injury. In the study, patients were randomly assigned to either the control group, which received standard Western medical treatment (including lung-protective ventilation strategies), or the experimental group, which received the same standard treatment supplemented with the TCM formula. The results indicated that following treatment, the experimental group exhibited marked improvements in oxygenation-related parameters, including heart rate, arterial partial pressure of oxygen, and Sao_2_ levels. Additionally, biomarkers such as white blood cell count, C-reactive protein, and procalcitonin showed notable reductions, while levels of inflammatory cytokines—including TNF-α, IL-6, and IL-8—decreased significantly. These findings provide strong evidence supporting the clinical application of the qi-tonifying, turbidity-resolving, and blood-circulation-promoting formula in the management of acute lung injury (Ren, 2020).

##### 4.2.1.4 Tongfu Xiefei Formula

A study involving 40 patients with acute lung injury caused by phlegm-heat congestion syndrome revealed that Tongfu Xiefei Formula could effectively reduce the partial pressure of carbon dioxide (Paco_2_), significantly increase the oxygenation index, and simultaneously decrease levels of DAO, MDA, NO, and inflammatory cytokines such as TNF-α and IL-6, thereby demonstrating a therapeutic effect on acute lung injury (Cheng, 2020).

##### 4.2.1.5 Qingwen Badu Yin

A randomized controlled trial conducted on 70 patients indicated that the addition of Qingwen Badu Yin to conventional treatment resulted in increased arterial partial pressure of oxygen (Pao_2_) and oxygenation index (Pao_2_/Fio_2_) compared to baseline measurements. Additionally, Paco_2_ levels were reduced after treatment, with more pronounced improvements observed in the treatment group (*P* < 0.05). Furthermore, serum levels of PCT, IL-6, TNF-α, and CRP were significantly decreased, and scores on the SOFA, APACHE II, and Murray lung injury scales were notably improved (Xu A. P. et al., 2024).

##### 4.2.1.6 Shengjiang Lifei Decoction

In a randomized controlled trial involving 60 patients, the treatment group received Shengjiang Lifei Decoction in conjunction with standard Western medical therapy. Following a seven-day treatment period, both groups exhibited improvements in Pao_2_, PaCo_2_, and oxygenation index compared to pre-treatment values, with greater therapeutic effects observed in the treatment group. Moreover, the duration of mechanical ventilation and ICU length of stay were shorter in the treatment group than in the control group. No adverse reactions were reported in either group throughout the treatment period, confirming that Shengjiang Lifei Decoction can enhance pulmonary function and alleviate clinical symptoms in patients with sepsis-induced ARDS, with favorable safety profile (Li G. C. et al., 2019).

#### 4.2.2 Clinical application of Traditional Chinese Medicine injections

##### 4.2.2.1 Shenfu Injection

In a randomized controlled trial involving 50 patients with acute lung injury (ALI), treatment with Shenfu Injection led to a significant reduction in levels of inflammatory markers, including C-reactive protein (CRP), interleukin-6 (IL-6), and tumor necrosis factor-alpha (TNF-α). Additionally, prothrombin time was shortened, and D-dimer levels were markedly decreased compared to baseline (Zhang et al., 2022).

##### 4.2.2.2 Xuebijing Injection

As one of the most widely prescribed traditional Chinese medicine (TCM) injections in current clinical practice, Xuebijing Injection has demonstrated notable therapeutic efficacy in managing ALI. Clinical evidence indicates that following intervention with Xuebijing Injection, multiple clinical and physiological parameters showed significant improvement. These include Traditional Chinese Medicine syndrome scores, Acute Physiology and Chronic Health Evaluation II (APACHE II) scores, Sequential Organ Failure Assessment (SOFA) scores, Murray Lung Injury scores, plateau pressure, and driving pressure (ΔP). Furthermore, levels of inflammatory biomarkers such as C-reactive protein, procalcitonin, interleukin-6, heparin-binding protein, white blood cell count, neutrophil count, neutrophil-to-lymphocyte ratio, and D-dimer were significantly reduced. Concurrently, pulmonary function indices—including arterial oxygen partial pressure (Pao2), oxygenation index (Pao2/Fio2), and static lung compliance—as well as immune function indicators such as CD3^+^, CD4^+^, and the CD4+/CD8+ ratio were notably elevated (Ding et al., 2024).

##### 4.2.2.3 Reduning Injection

Clinical investigations have revealed that Reduning Injection can substantially enhance cellular immune function in patients. The treatment group exhibited higher levels of CD3^+^, CD4^+^, and CD4+/CD8+ compared to the control group. The short-term overall effective rate in the treatment group reached 90.91%, significantly surpassing the 68.18% observed in the control group. More importantly, during a six-month follow-up period, the survival rate in the treatment group was 67.50%, compared to 56.67% in the control group, indicating sustained clinical benefits (Li et al., 2020).

#### 4.2.3 Clinical applications of monomers derived from Traditional Chinese Medicine and their derivatives

TCM monomers and their derivatives are diverse in nature, with complex structures and a broad range of pharmacological activities. Recent studies have increasingly highlighted the significant potential of these monomers and their combinations (i.e., component-based TCM) in treating ALI, offering safer and more effective therapeutic options for clinical use. While several reviews have extensively explored the mechanisms and clinical applications of TCM monomers in preventing and treating ALI, this paper aims to provide a deeper analysis by focusing specifically on the clinical applications of these monomers and their derivatives.

Although most studies on the efficacy of TCM monomers in treating ALI is still in the preclinical stage, their therapeutic potential is being reevaluated for both prevention and treatment, whether as individual monomers or in synthetic formulations. The primary objective of this section is to investigate the mechanisms through which these TCM monomers act in ALI treatment, thus laying a scientific foundation for their future clinical applications. Several TCM monomers have shown promising results in treating ALI, demonstrating their potential to modulate immune responses, reduce inflammation, and improve lung function.

##### 4.2.3.1 Ligustrazine

This compound has been shown to reverse macrophage polarization, reduce pyroptosis, and exert anti-ALI effects (Jiang R. et al., 2021). Clinical studies indicate that tetramethylpyrazine injection, which contain ligustrazine, significantly improve patients’ Pao_2_/Fio_2_ ratios and respiratory rates. It also reduces systemic inflammatory response syndrome (SIRS) and lowers APACHE II scores, as along with decreasing levels of IL-6, C-reactive protein (CRP), nitric oxide (NO). These effects help alleviate ALI symptoms and promote recovery (Liu et al., 2019).

##### 4.2.3.2 Diammonium glycyrrhizinate

This compound mitigates LPS-induced ALI by improving vascular endothelial barrier function (Liu et al., 2021). When combined with Astragalus injection, diammonium glycyrrhizinate significantly increases Po_2_/Fio_2_ ratios in patients, while also markedly reducing lactate (Lac), CRP, lung injury scores, APACHE-Ⅱ scores, and SIRS scores (Li et al., 2020).

##### 4.2.3.3 Anisodamine

Preclinical studies suggest that anisodamine inhibits inflammasome activation, thereby suppressing macrophage pyroptosis (Zhang et al., 2024). In a clinical study involving 78 patients who developed ALI after lung cancer surgery, intravenous administration of anisodamine over 48 h reduce the levels of TNF-α, IL-6, high-sensitivity C-reactive protein (hs-CRP), and procalcitonin (PCT). Additionally, it improves lung compliance, Pao_2_, and oxygenation indices, demonstrating its protective effects against ALI following thoracotomy for lung cancer (Du et al., 2017).

##### 4.2.3.4 Diacerein

Through randomized, double-blind clinical trials, it was demonstrated that Diacerein can inhibit the secretion of IL-1β on the surface of human neutrophils during viral replication and downregulate the expression levels of NLRP3, Caspase-1, and GSDMD (Carmo et al., 2024). According to the World Clinical Trial Database, a study involving 40 patients with COVID-19 was conducted using diacerein as an intervention. Serum samples collected 10 days after treatment were analyzed for changes in biomarkers such as IL-1β, IL-8, IL-6, C-reactive protein and troponin. However, the results of this trial have not yet been disclosed (Fundação de Amparo à Pesquisa do Estado de São, 2022). Further details are provided in Table 4.

**TABLE 4:** Clinical applications of monomeric components in the treatment of ALI.

Monomer species	CAS	Molecular formula	Case	Appropriate clinical evaluation metrics	Clinical research
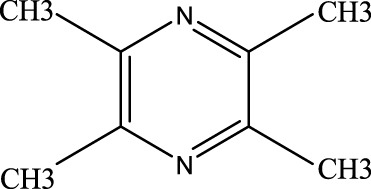 Ligustrazine	1124–11-4	C_8_H_12_N_2_	55	SIRS, APACHEⅡ score, IL-6, C-protein, NO↓	Ligustrazine can decrease the incidence of lung injury and improve patients’ respiratory rates
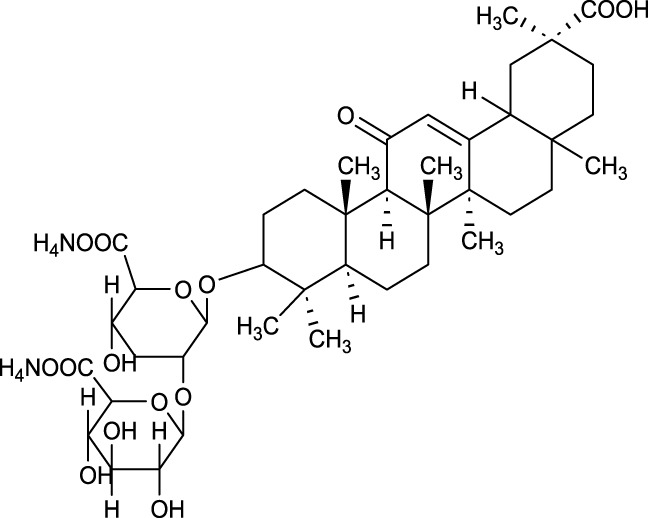 Diammonium glycyrrhizinate	79165–06-3	C_42_H_65_NO_16_	60	Lac, CRP, lung injury score, APACHE-Ⅱ, SIRS↓	Diammonium glycyrrhizinate can enhance the PO2/FiO2 ratio while reducing lactate (Lac), C-reactive protein (CRP), lung injury score, APACHE-II score, and SIRS score
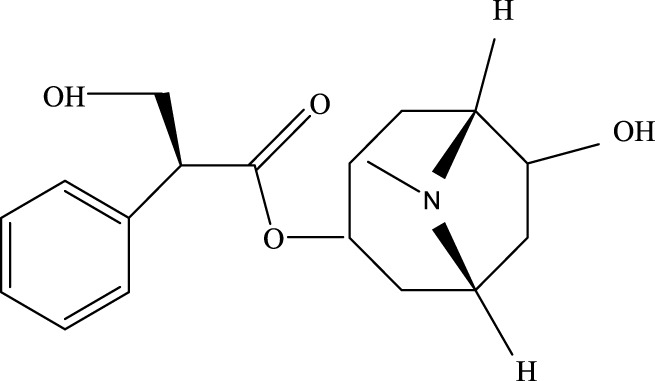 Anisodamine	55869–99-3	C_17_H_23_NO_3_	78	TNF-α, IL-6, hs-CRP, PCT↓	Anisodamine reduces the expression of hs-CRP, and PCT while enhancing lung compliance, PaO2 levels, and the oxygenation index
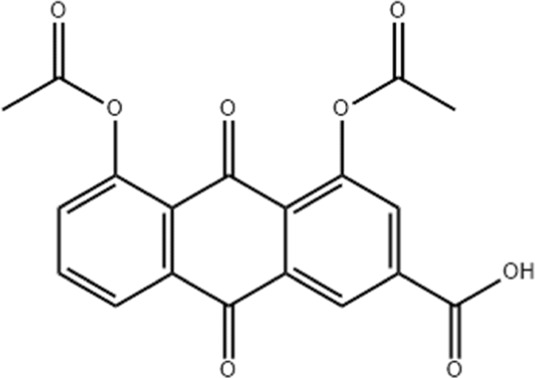 Diacerein	13739–02-1	C_19_H_12_O_8_	40	IL-1β, IL-6, IL-8, C-reactive protein	/

## 5 Discussion

According to the theory of Traditional Chinese Medicine (TCM), acute lung injury (ALI) can be categorized under syndromes such as “asthma syndrome” and “sudden asthma”. Its etiology primarily involves pathological changes including deficiency of vital energy and accumulation of pathogenic toxins in the lungs. Consequently, clinical treatment strategies emphasize replenishing qi, nourishing Yin, clearing heat, and detoxifying (Xie et al., 2008). The pathogenesis of ALI involves a complex interplay of multiple pathophysiological mechanisms. Key pathogenic processes include excessive inflammatory response, disruption of the alveolar-capillary barrier, oxidative stress, and apoptosis. During the initial phase of lung injury—triggered by factors such as infection, trauma, or aspiration—pathogen-associated molecular patterns (PAMPs) or damage-associated molecular patterns (DAMPs) activate alveolar macrophages and neutrophils, resulting in the release of substantial amounts of pro-inflammatory cytokines, such as TNF-α, IL-1β, and IL-6. These cytokines further recruit additional immune cells, leading to an inflammatory cascade (Vora et al., 2021). Concurrently, activated neutrophils secrete proteases, including elastase, along with reactive oxygen species (ROS), which directly damage alveolar epithelial and vascular endothelial cells. This results in the degradation of tight junction proteins, such as ZO-1 and Occludin, increased vascular permeability, and the development of pulmonary edema (Yu L. C. et al., 2024). Moreover, dysfunction and apoptosis of alveolar type II epithelial cells (AT2) impair surfactant production, thereby exacerbating alveolar collapse. Recent studies have also indicated that cellular metabolic reprogramming, mitochondrial dysfunction, endoplasmic reticulum stress, and regulated cell death pathways like ferroptosis and pyroptosis are not mere consequences but are central drivers of ALI progression, modulating apoptotic and autophagic pathways (Wang et al., 2025; Taheri et al., 2024). These mechanisms interact synergistically, forming a self-amplifying cycle that culminates in impaired gas exchange and respiratory failure.

Although significant progress has been made in the clinical and experimental research on TCM for ALI, there are still many issues worthy of in-depth exploration. Firstly, the quintessential strength of TCM lies in its holistic regulation and personalized treatment based on syndrome differentiation. Therapeutic principles such as clearing heat and resolving phlegm, or promoting blood circulation to unblock meridians, do not merely suppress excessive inflammatory responses but also aim to improve microcirculation and promote tissue repair. This multi-target, systematic approach contrasts sharply with the single-target interventions often prioritized in Western medicine. However, a critical gap remains: most current studies focuses on the mechanisms of single herbs or monomer components, while the synergistic effects arising from the complex interplay of components within a formula—governed by the principles of “sovereign, minister, assistant, and messenger” — are poorly understood. Bridging this gap is essential to elucidating the material basis of TCM efficacy. Secondly, while the anti-inflammatory and antioxidant mechanisms of certain TCM monomers (such as artemisinin and tanshinone IIA) have been well-characterized, translating these findings into clinical practice requires overcoming significant hurdles, such as optimizing dosage for a precise therapeutic window that balances efficacy with safety, and developing novel drug delivery systems to improve bioavailability at the target site.

The rapid evolution of multi-omics technologies presents a paradigm-shifting opportunity for TCM pharmacology. An integrated multi-omics analysis, combining genomics, transcriptomics, proteomics, and metabolomics, can provide an unbiased, panoramic view of the body’s response to TCM interventions. This approach allows us to move beyond a one-molecule-one-target framework and instead map the complex interaction network through which TCM components modulate the genome, transcriptome, and proteome, thus revealing the multi-level regulatory landscape of TCM. Network pharmacology, as a computational method, further helps to deconstruct this complexity by identifying core targets and pathways, providing a robust theoretical basis for clinical applications (Wang Z. Y. et al., 2022). Furthermore, the combination of cutting-edge technologies, such as single-cell technology and spatial omics technology, has brought new opportunities and challenges to dissect the mechanism of TCM in modulating cellular and spatial heterogeneity (Chen et al., 2022). Through these technologies, we can pinpoint how specific TCM formulas regulate distinct cell populations (e.g., macrophage subtypes, endothelial cells) within the lung microenvironment and understand the spatial distribution of key proteins, thereby offering a more comprehensive explanation of TCM’s therapeutic mechanisms. This is a key direction for future work.

TCM compound prescriptions are the cornerstone of clinical TCM practice. However, due to the complex chemical composition of medicinal materials used in these formulations and the influence of multiple factors—including the origin of raw materials, processing methods of decoction pieces, and production techniques—the quality control of TCM faces significant challenges, often summarized as “uncertain efficacy and imprecise dosage”. This has become a major bottleneck for modernization, industrialization, and internationalization of TCM (Sun et al., 2017). Therefore, it is imperative to establish a multi-dimensional quality control system. This system should integrate qualitative and quantitative dual-standards, spectroscopic-effect relationship analyses, network pharmacology- and metabolomics-based identification of quality markers (Q-markers), and the combination of multi-component quantification with bioactivity assays. Only through such a comprehensive quality evaluation system can we ensure the consistency and reliability of TCM preparations (Lu et al., 2024).

In the management of ALI, an integrative model combining TCM and Western medicine holds immense untapped potential. Western medicine provides objective, quantifiable biomarkers and rapid, life-sustaining interventions. In contrast, TCM emphasizes individualized treatment and holistic regulation, which can be highly beneficial for alleviating symptoms and improving quality of life (Yu et al., 2023). However, a significant challenge lies in reconciling their different diagnostic and therapeutic philosophies within the rigid framework of conventional randomized controlled trials (RCTs). The “one-size-fits-all” design of RCTs often conflicts with the personalized nature of TCM syndrome differentiation, potentially masking the true efficacy of a formula when applied to a heterogeneous patient population. Thus, future clinical trial designs should incorporate biomarker-guided patient stratification or adaptive designs that allow for treatment adjustments based on TCM pattern diagnosis. Merging TCM’s holistic and individualized approach with Western medicine’s precise and emergency care models represents the most promising path forward for optimizing patient outcomes in ALI (Li et al., 2025).

Finally, conventional pharmacokinetics, which focuses on systemic drug exposure, often overlooks the critical role of the gut. Many TCM components have low oral bioavailability and exert their effects by modulating the gut microbiota or being transformed into active metabolites by it. Therefore, it is essential to investigate the “substance-effect” relationship within the intestinal lumen, clarifying how TCM components and their gut microbial metabolites interact to produce therapeutic effects or adverse reactions. This will provide a more complete picture of the multi-target interaction profiles of TCM and enhance the safety and efficacy of its clinical application (Zhu et al., 2024).

## 6 Conclusion

In conclusion, this review has systematically summarized the multifaceted pathogenesis of ALI and highlighted the significant therapeutic potential of TCM, acting through multi-component, multi-target, and multi-pathway mechanisms. While promising, the translation of TCM from bench to bedside requires a concerted effort to address existing challenges. Future efforts should prioritize the accumulation of high-quality clinical evidence through innovatively designed multicenter, international collaborations to achieve broader recognition from the mainstream medical community. Clinically, integrating TCM syndrome differentiation with modern diagnostic biomarkers can enable a more personalized and precise application of therapies, creating a synergistic effect that offers more comprehensive treatment for ALI patients. Mechanistically, the application of multi-omics and systems biology approaches is crucial for elucidating the complex pharmacological basis of TCM’s efficacy. Additionally, exploring the novel therapeutic potential of existing TCM components and strengthening evidence-based research on integrative medicine will not only elevate therapeutic outcomes but also enhance the scientific rigor and validation of TCM, providing a more reliable foundation for clinical decision-making in the fight against ALI.
